# Racial and Sex Differences between Urinary Phthalates and Metabolic Syndrome among U.S. Adults: NHANES 2005–2014

**DOI:** 10.3390/ijerph18136870

**Published:** 2021-06-26

**Authors:** Rajrupa Ghosh, Mefruz Haque, Paul C. Turner, Raul Cruz-Cano, Cher M. Dallal

**Affiliations:** 1 Department of Epidemiology and Biostatistics, School of Public Health, University of Maryland, College Park, MD 20742, USA; rajrupa@terpmail.umd.edu (R.G.); haque.mefruz@gmail.com (M.H.); raulcruz@umd.edu (R.C.-C.); 2 Maryland Institute for Applied Environmental Health, School of Public Health, University of Maryland, College Park, MD 20742, USA; pturner3@umd.edu

**Keywords:** phthalates, metabolic syndrome (MetS), race, sex

## Abstract

Phthalates, plasticizers ubiquitous in household and personal care products, have been associated with metabolic disturbances. Despite the noted racial differences in phthalate exposure and the prevalence of metabolic syndrome (MetS), it remains unclear whether associations between phthalate metabolites and MetS vary by race and sex. A cross-sectional analysis was conducted among 10,017 adults from the National Health and Nutritional Examination Survey (2005–2014). Prevalence odds ratios (POR) and 95% confidence intervals (CIs) were estimated for the association between 11 urinary phthalate metabolites and MetS using weighted sex and race stratified multivariable logistic regression. Higher MCOP levels were significantly associated with increased odds of MetS among women but not men, and only remained significant among White women (POR _Q4 vs. Q1_ = 1.68, 95% CI: 1.24, 2.29; *p*-trend = 0.001). Similarly, the inverse association observed with MEHP among women, persisted among White women only (POR _Q4 vs. Q1_ = 0.53, 95% CI: 0.35, 0.80; *p*-trend = 0.003). However, ΣDEHP metabolites were associated with increased odds of MetS only among men, and this finding was limited to White men (POR _Q4 vs. Q1_ = 1.54, 95% CI: 1.01, 2.35; *p*-trend = 0.06). Among Black men, an inverse association was observed with higher MEP levels (POR _Q4 vs. Q1_ = 0.43, 95% CI: 0.24, 0.77; *p*-trend = 0.01). The findings suggest differential associations between phthalate metabolites and MetS by sex and race/ethnicity.

## 1. Introduction

Phthalates are found in a wide range of household, consumer, and personal care products [1]. Low molecular weight (LMW) phthalates, including diethyl phthalate (DEP) and dibutyl phthalate (DBP), are used as solvents in personal care products such as fragrances, lotions, and cosmetics, as well as in pharmaceutical coatings [1,2], whereas high molecular weight (HMW) phthalates, including Di(2-ethylhexyl) phthalate (DEHP) metabolites, are commonly used as plasticizers in food packaging materials, consumer products, and medical devices [1,2]. Plasticizers are able to leach into products or the environment, facilitating human exposure via ingestion, inhalation, and dermal contact [1,3]. Given the multiple and widespread use of phthalates, the majority of the US population is continuously exposed [2]. 

Women and racial ethnic minorities (Black and Mexican/Hispanic individuals) are reported to have higher urinary levels of phthalate metabolites than their White counterparts [4,5,6]. Increased use of personal care products, such as cosmetics, fragrances, and hair care products, may explain the higher phthalate metabolite levels among women [7,8]. Potential explanations for this include increased frequency of specific hair care [9] and feminine hygiene product [4] use among Black women, and personal care product use [10] among Latina women.

Phthalates are classified as xenoestrogens and have been negatively associated with several health conditions [11], including metabolic disorders such as obesity and insulin resistance [12,13,14,15]. The association between phthalate exposure and dyslipidemia is inconsistent, with two studies reporting either positive [16] or null associations [17]. These disorders contribute to metabolic syndrome (MetS), defined as having at least three of the five following criteria: elevated abdominal obesity, elevated triglycerides, high blood pressure, elevated fasting glucose levels, and reduced high-density lipoprotein (HDL) levels [18]. MetS affects about 35% of US adults [19], with noted differences by sex and race, and is associated with an increased risk of several chronic diseases, including cardiovascular diseases [20], diabetes [21], and cancer [22].

Peroxisome proliferator–activated receptors (PPARs), functionally activated by both a lower or higher concentration of phthalates, play a critical role in adipogenesis and lipid storage [23,24,25]. The interaction of phthalates with PPARs affects glucose metabolism and leads to adipogenesis and disturbs metabolic homeostasis, subsequently resulting in metabolic dysfunctions [23,24,25]. Hence, the alteration of the lipid and carbohydrate cell signaling pathways has been hypothesized as a mechanism of action for phthalates.

While prior studies have investigated the relationship between urinary phthalates and individual components of MetS [12,14,16,26], only one study to date has investigated associations between urinary phthalate concentrations and MetS among US adults [16]. This prior study included participants from the National Health and Nutritional Examination Survey (NHANES) cycles 2001–2010, and observed sex differences in associations between specific urinary phthalate metabolites and MetS [16]. However, despite evidence of racial/ethnic disparities in both phthalate exposure and the prevalence of MetS [4,5,19], it remains unclear whether racial differences exist in the association between phthalate metabolites and MetS. The objective of this analysis was to assess whether phthalate metabolite concentrations are differentially associated with MetS among Black, Mexican/Hispanic, and White individuals who participated in NHANES cycles 2005–2014.

## 2. Materials and Methods

Study Design and Population: This cross-sectional analysis utilized data from the National Health and Nutrition Examination Survey (NHANES) across five survey year cycles conducted during 2005–2014. NHANES is a nationally representative survey administered by the Centers for Disease Control and Prevention National Center for Health Statistics (NCHS) [27,28]. It uses a complex, multistage probability sampling strategy that oversamples certain subgroups of the non-institutionalized civilian resident population of the United States, residing in the 50 states and in Washington D.C. [27,28]. Additional details on NHANES are published elsewhere [27,28].

The initial analytic population consisted of 16,264 individuals with data on phthalate metabolites during the 2005–2014 survey year cycles. Urinary phthalate metabolites were measured by NHANES in a randomly selected subsample in each survey cycle. Participants aged <18 years (n = 4610) were excluded from the analysis, as the criteria for classifying metabolic syndrome varies for children [29]. Additionally, the following exclusions were applied: participants undergoing dialysis (n = 44), those who reported being pregnant or were lactating (n = 301), those with missing values for phthalate metabolites (n = 261), observations with a sample weight = 0 (n = 124), and individuals who selected “other race-including multiracial” (n = 907) due to heterogeneity. The final analytic sample consisted of 10,017 adults ≥ 18 years, for whom urinary phthalate measurements were available for the 2005–2014 survey years.

Data Collection and Covariate Information: Data on demographics, 24-h dietary intake (total caloric intake (kcal)), and smoking status were collected via questionnaires through in-person interviews. The family poverty–income ratio (PIR) compares family income to US census-defined poverty levels, with a value of 1.0 indicating the federal poverty threshold and above 1.0 indicating income above poverty. Smoking status (current/former/never) was determined via responses to two questions regarding having smoked at least 100 cigarettes in their lifetime (yes/no) and their current smoking status (yes/no).

Blood collection and physical measurements were taken in the mobile examination center by trained health technicians, including height, weight, waist circumference, and blood pressure, following standard NHANES procedures. A random subsample of NHANES participants were asked to fast for ≥8 h prior to blood collection. Fasting status was documented using a questionnaire prior to blood draw. Fasting samples were used to measure the blood glucose and serum triglycerides. High blood pressure (yes/no) was defined by either an average of three consecutive blood pressure measurements or if current blood pressure medication use was indicated on the interview questionnaire. High blood glucose (yes/no) was defined by either plasma glucose levels or if current insulin/diabetes medication use was indicated in the interview questionnaire. Details on standardized NHANES procedures have been previously described [30,31,32,33,34].

Measurement of exposure: Phthalate metabolites were measured in spot urine samples using high performance liquid chromatography-electrospray ionization-tandem mass spectrometry (HPLC-ESI-MS/MS), as previously described [30]. The specific phthalate metabolites measured varied across the survey years, with a total of 16 phthalate metabolites measured across the 2005–2014 survey years. Phthalate metabolites were retained in this analysis if (i) the metabolite was measured in all of the survey cycle years, (ii) less than 50% of the values for a specific metabolite were missing across the survey cycle years, and (iii) more than 50% of the samples were at or above the analytical limit of detection (LOD).

The following 11 phthalate metabolites were retained: mono-ethyl phthalate (MEP), mono-n-butyl phthalate (MnBP), mono-isobutyl phthalate (MiBP), mono carboxynonyl phthalate (MCNP), mono carboxyoctyl phthalate (MCOP), mono-(3-carboxypropyl) phthalate (MCPP), and mono-benzyl phthalate (MBzP), and the di(2-ethylhexyl) phthalate (DEHP) metabolites of mono-(2-ethyl-5-carboxypentyl) phthalate (MECPP), mono-(2-ethyl-5-hydroxyhexyl) phthalate (MEHHP), mono-2-ethylhexyl phthalate (MEHP), and mono-(2-ethyl-5-oxohexyl) phthalate (MEOHP). Different limits of detection (LOD) were used by NHANES across the survey cycles (see Table S1). To estimate the LOD in this analysis, the highest reported LOD for any given metabolite across all survey years was used. The proportion of samples below the LOD was less than 10% for all of the metabolites, with the exception of MEHP (40%) (Table S1). The concentration of metabolites below the LOD were replaced with the LOD for that metabolite divided by the square root of two [35]. In addition to analyzing the individual metabolites, the following summary measures were generated and analyzed: low molecular weight (ΣLMW) metabolites (MEP + MnBP + MiBP), DEHP (ΣDEHP) metabolites (MEHP + MECPP + MEHHP + MEOHP), and high molecular weight (ΣHMW) metabolites (MCNP + MCOP + MCPP + MBzP + ΣDEHP).

Measurement of outcome: Metabolic syndrome (MetS) was defined using the National Cholesterol Education Program’s Adult Treatment Panel III report criteria [18]. Participants were classified as having MetS if they met at least three of the following five criteria: waist circumference ≥102 cm (men) or ≥88 cm (women), fasting serum triglycerides ≥150 mg/dl, high density lipid (HDL) cholesterol <40 mg/dl (men) or <50 mg/dl (women), blood pressure ≥130/85 mm Hg or treatment for hypertension, and fasting blood glucose ≥ 100 mg/dl or treatment for diabetes [18]. Among those with MetS (n = 2403), 68% had three, 24.4% had four, and 7.6% had all five of the MetS components. The majority of those with MetS had a high waist circumference (93.1%), followed by high blood pressure (83.6%) and low HDL levels (70.6%). Individuals were not excluded if data on specific MetS components were missing, given that Mets is diagnosed based on the presence of a minimum of three components. In the overall population, the availability of MetS component data was as follows: waist circumference (98.3%), fasting serum triglycerides (46.0%), HDL (98.0%), high blood pressure (97.4%), and fasting blood glucose (50.2%). As fasting samples were only collected in a subsample of the NHANES population, glucose and triglyceride status was unknown for 49.8% and 54.0% of the population, respectively. However, among those who were classified as not having MetS in our analytic population but had met two of the criteria above (n = 2702), 20.2% were missing glucose and 21.4% triglycerides.

Statistical Analysis: Distributions of the demographic, behavioral, and metabolic variables were assessed by estimating the frequencies and weighted percentages (categorical variables) and weighted means and standard errors (continuous variables). Distributions were examined by sex and race, and further by the presence of MetS status. Independent sample *t*-tests (continuous variables) and chi-square tests (categorical variables) were utilized for bivariate analyses to evaluate race and sex differences. When used as a continuous variable, individual phthalate metabolites, ΣLMW, ΣHMW, and ΣDEHP were log (natural log) transformed to account for non-normal right skewed data. Weighted geometric means (GM) and 95% confidence intervals (CI) were estimated for the levels of individual phthalate metabolites, ΣLMW, ΣHMW, and ΣDEHP for the following analytic populations: overall, sex stratified, and race and sex stratified. Correlations between the phthalate metabolites were estimated by Spearman correlation coefficients. MetS was analyzed as a dichotomous outcome, while phthalate metabolites or sums of metabolites were categorized based on quartile distributions in the overall population. Multivariable logistic regression models were used to estimate the prevalence odds ratios (POR) and 95% confidence limits (CI) for the association between urinary phthalate metabolites and MetS overall, as well as stratified by (1) sex and (2) sex and race.

Creatinine was included as a covariate in all models to adjust for urine concentration and flow rate. Based on the existing literature, the following a priori potential confounders were included in the final models: age (continuous), race (White/Black/Hispanic or Mexican), sex (male/female), education (<high school/ ≥ high school/college graduate/unknown), poverty status (at or above/below poverty level/unknown), fasting status (fasted for ≥8 h/fasted for <8 h/unknown), daily calorie intake (tertiles), and smoking status (never/former/current/unknown). To retain the observations with missing values in the logistic regression models, an unknown category was included (refusals, do not know, and missing responses). Tests for trend were performed by modeling exposure to phthalate metabolites as an ordinal variable. Because of the multistage probability sampling of NHANES, the sample weights were used in all analyses to ensure the representativeness of the US adult population. Based on the NHANES analytic guidelines [36], a new sample weight was constructed for the combined survey years of 2005–2014 by dividing the 2-year weights for each cycle by 5. Bonferroni corrections for multiple comparisons were not applied. The statistical significance of all analyses was assessed at α= 0.05. All analyses were conducted using SAS 9.4 (Cary, NC, USA).

## 3. Results

The overall study population consisted of a nearly equal proportion of men and women, n = 5060 (49.6%) and 4957 (50.4%), respectively. Among men (Table 1), White men were older, more educated, with a higher socioeconomic status, and had lower BMI compared with Black and Mexican/Hispanic men. Levels of serum triglycerides and glucose were the highest among Mexican/Hispanic men, whereas high blood pressure and smoking were more common among Black men. The prevalence of MetS was the highest among White men (21.8%). When stratified further by MetS status, those with MetS had a higher average age and BMI across all races when compared with those without MetS (Table S2). Among women (Table 2), White women were slightly older, more educated, with a higher socioeconomic status, and higher proportion of current smokers compared with Black and Mexican/Hispanic women. Black women had a higher BMI, calorie intake, waist circumference, and blood pressure compared with both White and Mexican/Hispanic women. The prevalence of MetS was the highest among Black women (23.6%). Similar to men, women of all races with MetS had a higher average age and BMI compared with women without MetS (Table S3). Among those with MetS, Black men and women had the highest blood pressure, while Mexican/Hispanic men and women had the highest serum triglyceride levels (Tables S2 and S3).

Geometric means (GM) of urinary phthalate metabolite concentrations (ng/mL) among the overall population were the highest for MEP (GM = 71.1, 95% CI: 67.1, 75.3), MECPP (GM = 20.6, 95% CI: 19.3, 22.1), MEHHP (GM = 13.4, 95% CI: 12.5, 14.4), and MnBP (GM = 12.8, 95% CI: 12.1, 13.5), and the lowest for MEHP (GM = 1.7, 95% CI: 1.6, 1.8) (Table S1). When stratified by sex, men had slightly higher levels compared with women (Table S4). Correlations between all phthalate metabolites were positive and statistically significant (*p* < 0.0001) (Table S5). The strongest correlations were observed between MEOHP and MEHHP (r = 0.98), MEHHP and MECPP (r = 0.94), MEOHP and MECPP (r = 0.94), MEHHP and MEHP (r = 0.78), and MEOHP and MEHP (r = 0.77).

Higher levels of most phthalate metabolites were observed among Black men compared with White and Mexican/Hispanic men (Table 3). A similar pattern was noted among women, where Black women had the highest levels of all urinary phthalate metabolites, followed by Mexican/Hispanic women (Table 3). The levels of summary metabolites (∑LMW, ∑DEHP, and ∑HMW) were higher among Black men and women, followed by their Mexican/Hispanic and White counterparts (Table 3).

In the overall multivariable analysis, higher MCOP and MECPP levels were significantly associated with an increased odds of the prevalence of MetS (MCOP: POR _Q4 vs. Q1_ = 1.25, 95% CI: 1.02, 1.54; *p*-trend = 0.06; MECPP: POR _Q4 vs. Q1_ = 1.26, 95% CI: 1.01, 1.59; *p*-trend = 0.03) (Table S6). Conversely, higher MEHP levels were inversely associated with the odds of MetS prevalence (POR _Q4 vs. Q1_ = 0.71, 95% CI: 0.55, 0.90; *p*-trend = 0.02) (Table S6). When stratified by sex (Table S7), MCOP was positively associated with the odds of MetS prevalence only among women (POR _Q4 vs. Q1_ = 1.51, 95% CI: 1.17, 1.95; *p*-trend = 0.002), while higher levels of MECPP were associated with MetS among men (POR _Q4 vs. Q1_ = 1.40, 95% CI: 0.99, 1.98; *p*-trend = 0.02). Higher levels of MEHHP (POR _Q4 vs. Q1_ = 1.39, 95% CI: 1.04, 1.86; *p*-trend = 0.04), as well as MEOHP (POR _Q4 vs. Q1_ = 1.48, 95% CI: 1.09, 2.01; *p*-trend = 0.02), were associated with MetS among men but not among women. The inverse association between MEHP with the odds of MetS persisted, but was only observed among women (POR _Q4 vs. Q1_ = 0.54, 95% CI: 0.38, 0.76; *p*-trend = 0.001) (Table S7).

Findings from the sex and race stratified analysis are presented in Table 4 and Table 5. An inverse association between MEP and the odds MetS prevalence was observed among Black men (POR _Q4 vs. Q1_ = 0.43, 95% CI: 0.24, 0.77; *p*-trend = 0.01) (Table 4). Among women (Table 5), increasing urinary levels of MCOP were significantly associated with the odds of Mets prevalence among White women only (POR _Q4 vs. Q1_ = 1.68, 95% CI: 1.24, 2.29; *p*-trend = 0.001). An inverse association between MEHP and the odds of MetS prevalence was also observed only among White women (POR _Q4 vs. Q1_ = 0.53, 95% CI: 0.35, 0.80; *p*-trend = 0.003) (Table 5). No other statistically significant threshold or dose–response relationships were observed in the sex and race stratified analyses.

In the sex stratified models of the summary measures (Table S9), ∑DEHP was positively associated with the odds of MetS prevalence among men (POR _Q4 vs. Q1_ = 1.57, 95% CI: 1.13, 2.17; *p*-trend = 0.01) but not women. When stratified by race and sex (Table 6 and Table 7), the association between ∑DEHP and an increased odds of MetS was observed among White men only (POR _Q4 vs. Q1_ = 1.54, 95% CI: 1.01, 2.35; *p*-trend = 0.06). An inverse relationship was observed between ∑LMW and the odds of MetS prevalence among Black men with a similar magnitude of effect across all quartiles (POR _Q4 vs. Q1_ = 0.42, 95% CI: 0.24, 0.75; *p*-trend = 0.02) (Table 6). Among women, both overall and by race, no significant patterns or dose–response relationships were observed with the summary metabolites.

## 4. Discussion

In this sex and race stratified cross-sectional analysis of NHANES cycles of 2005–2014, higher levels of MCOP were significantly associated with an increased odds of MetS among women, and, in particular, among White women. The association between higher MEHP levels and reduced odds of MetS was also restricted to White women. Among men, an inverse association was observed with elevated MEP levels, which was observed among Black men only, in the analyses stratified by race. Our analysis extended the prior sex-stratified NHANES analysis of phthalates and metabolic syndrome conducted by Todd et al. [16], specifically with regards to the additional stratification by race, survey cycles, and metabolites considered in our analysis. Todd et al. evaluated five metabolites (MEP, MnBP, MiBP, MBzP, and MCPP) along with the ∑DEHP metabolites (MEHP, MEHHP, and MEOHP). We included these metabolites along with three others (MCOP, MCNP, and MECPP) that met our conditions for inclusion in the analysis. In addition to the noted extensions, prior studies supporting racial/ethnic differences in phthalate exposure [4,5] and metabolic consequences, such as obesity [37,38] and diabetes [39,40], warranted the exploration of racial/ethnic differences in associations between urinary phthalate metabolites and the prevalence of metabolic syndrome. Despite the differences in the methods (inclusion/exclusion criteria and survey cycles) to derive analytic populations between the present study and that of Todd et al., we too observed an increased odds of MetS with higher levels of ∑DEHP among men only, and our analyses further revealed that this finding was restricted to White men. Todd et al. observed a significant association between higher levels of MBzP and increased odds of MetS, mainly among women, whereas in our analysis, significant findings were restricted to only one quartile among men, particularly among White men, with no support for threshold or dose–response relationships.

Prior studies have suggested higher phthalate metabolite concentrations among women [5,6] versus men, in part, due to the higher use of personal care products and cosmetics by women and, subsequently, the increased susceptibility to dermal phthalates exposure [7,8]. In our study, the levels of each phthalate metabolite were higher among men when stratified by sex. However, when stratified by race and sex, the highest levels of LMW metabolites were observed among black women, compared with men and women of other races. This finding may, in part, be explained by the increased use of specific personal care products [4,9], the primary sources of LMW phthalates [1]. Among men and women, higher levels of metabolites were generally observed among Black men and women, compared with Mexican/Hispanic and White men and women. Prior research suggests that phthalate exposure may be more prevalent among the non-Hispanic Black population in comparison with non-Hispanic White and Mexican Americans [5]. While we mostly observed higher urinary phthalate levels among Black men and women, statistically significant findings from our race and sex stratified analyses of phthalate metabolites and the prevalence of MetS did not reveal similar patterns across the metabolites. However, it is also important to note that while our sample included Black and Mexican/Hispanic men and women, 73% of our sample identified as White.

Changes in the chemical environment have been correlated with metabolic disorders [25] and, more specifically, prior studies have examined phthalate metabolites in relation to the individual components of Mets [12,14,26], with the majority focused on obesity [12,14,15] and diabetes [6,12,41]. Although comparatively limited, evidence also supports a positive association with hypertension [26,42]. Stahlhut et al. [14] and Hatch et al. [15] reported that several phthalate metabolites, including MBzP, MEHHP, MEOHP, and MEP were associated with an increased BMI and waist circumference [14,15]. Additionally, Huang et al. [6] and Todd et al. [41], using the NHANES 2001–2008 cycles, reported that higher levels of the metabolites MBzP, MnBP, MiBP, MCPP, and ∑DEHP were associated with both an increased odds [41] and increased risk [6] of diabetes, with differences in the strength of the dose–response relationships by race/ethnicity [6]. In our analysis, select quartiles of MiBP were associated with the odds of MetS among Mexican/Hispanic men and women; however, there was no evidence of a threshold or a dose–response relationship. Overall, with regards to MetS, epidemiological studies examining the role of phthalate metabolites are limited [16]. Findings from Todd et al. and the present analysis support associations with select phthalate metabolites and MetS, and warrant further investigation utilizing prospective studies, particularly with larger samples of individuals of different races/ethnicities, including Asian men and women, as well as other groups not represented in our analysis.

Phthalates are classified based on their molecular weight, either LMW or HMW [43], with differing toxicological properties and applications. LMW phthalates are more water soluble than HMW metabolites, enabling them to be excreted quickly as its primary metabolite, in contrast with HMW phthalates, which need to be oxidized further to their secondary metabolic form to be excreted [43]. As a result, HMW phthalates have a greater affinity to be stored longer than LMW phthalates. In our analysis, the HMW phthalates metabolites, including MCOP, and the DEHP metabolites (MECPP, MEHHP, and MEOHP), were associated with increased odds of MetS, while the DEHP metabolite, MEHP, was inversely associated with the odds of MetS. An inverse association was also observed between higher levels of ∑LMW phthalate metabolites and increased odds of MetS; this finding was observed only among Black men.

While we observed an inverse association between MEHP and odds of MetS, this finding was restricted to White women in the race and sex stratified analyses. The explanations for this somewhat surprising finding are unclear, but may involve MEHP metabolites associations with androgens. Obesity and MetS are associated with higher androgen levels among women [44], which, in turn, have been shown to be inversely associated with MEHP [45]. It is also important to note that MEHP metabolites had the highest proportion of values below the LOD (40%). Additionally, we cannot dismiss the possibility that this finding or others in our analysis may be due to chance or reverse causation.

In addition to the potential role of androgens, the activation of PPARs [24,25] has been proposed as a potential biological mechanism, whereby EDCs may influence MetS [25]. The activation of PPARs has an important role in adipogenesis, insulin sensitivity control, and inflammatory responses, all key aspects of MetS [23,25]. Phthalates and its metabolites can bind to and activate PPARs, and disrupt the metabolic pathways [25]. Additional pathways involve the inappropriate inactivation of hormonal receptors such as those of estrogen, thyroid, and glucocorticoid receptors [25]. These receptors can be targeted by EDCs, which then bind to them and initiate reproductive effects and metabolic alterations.

Limitations should be considered when interpreting these findings. While we were able to stratify our analyses by sex and race, 73% of the analytic population self-identified as White, warranting the need for future studies with more racial/ethnic variation in the study population. Our analysis utilized the cross-sectional design, precluding the ability to assess temporality and underlying etiologic hypotheses. Moreover, our analysis and the majority of prior studies relied on a single spot urine sample measurement of phthalate metabolites. Given the short biological half-lives (3–18 h) of phthalates [46], urinary metabolite levels only reflect exposures that occurred ≤1 day before the urine sample collection [47], and may not reflect long term exposure. Additionally, the timing and frequency of phthalate measurement is important given the changes in levels by sources such as diet, personal care products, medications [1,46]. Variation due to diet is especially relevant for HMW phthalates [1], including the DEHP metabolites, where the levels decrease with fasting times of ≥6 h [46,48]. Prior literature has also shown that the highest serum levels of specific phthalates were detected 2–4 h after the use of phthalate containing products, while the levels decreased after 24 h of use [49]. Hence, excluding participants based on their fasting time may affect the levels of urinary phthalate metabolites detected, potentially leading to exposure misclassification. To minimize this, participants with missing data on their fasting status were included, and the analyses were adjusted for whether an individual fasted or if their status was unknown. The inclusion of those with unknown fasting status subsequently resulted in missing values for glucose and triglyceride levels. However, as previously described, a diagnosis of MetS requires a minimum of three of the five components, precluding the need to restrict our analytic file based on the availability of all five components. While this approach was intended to be more conservative in terms of capturing individuals with MetS, it limited our ability to conduct component specific analysis, especially with regards to glucose and triglycerides. It also may have led to potential outcome misclassification and, specifically, an underestimation of MetS prevalence. The MetS in our analytic population was approximately 20% versus estimates of 35% in the general US population [19].

The strengths of this analysis include the focus on exploring racial differences in associations between 11 phthalate metabolites and MetS, including analyses of summary measures based on their molecular weight. Additionally, this study utilized clinical examination and laboratory data from NHANES to assess metabolic components in lieu of relying only on self-report, which often underestimates the prevalence of undiagnosed conditions such as hypertension and diabetes. Furthermore, NHANES includes a nationally representative sample and, thus, the results from the analysis are generalizable to the US population.

## 5. Conclusions

Our study points towards differential associations between select phthalate metabolite concentrations and the prevalence of MetS by race and sex. However, given the cross-sectional nature of the analysis, it is important to further evaluate the role of these metabolites with the risk of developing MetS in order to help understand the long-term effects of these chemicals on public health. In summary, the ubiquitous presence of phthalates in personal care and consumer products, its association with MetS, and the suggested differences in observed in sex and race stratified associations in this analysis warrants further studies, ideally using longitudinal designs with multiple time points and importantly, with study populations with racial/ethnic diversity.

## Figures and Tables

**Table 1:** Demographic and metabolic characteristics among men, National Health and Nutrition Examination Survey (NHANES) (2005–2014).

	White (n = 2503)	Black (n = 1211)	Mexican/Hispanic (n = 1346)
	Mean (SE) ^a^		
Age (years)	46.9 (0.4)	42.3 (0.5)	39.0 (0.4)
Body mass index (kg/m^2^)	28.8 (0.1)	29.2 (0.2)	29.1 (0.2)
Total calorie intake (Kcal)	2623 (26)	2503 (39)	2561 (38)
Urinary creatinine (mg/dl)	136.8 (1.8)	184.0 (3.1)	144.5 (2.4)
Waist circumference (cm)	102.4 (0.4)	98.8 (0.6)	99.9 (0.5)
Systolic blood pressure (mm Hg)	123.8 (0.4)	127.3 (0.6)	121.6 (0.4)
Diastolic blood pressure (mm Hg)	71.8 (0.3)	72.8 (0.4)	70.0 (0.4)
Serum triglycerides (mg/dl)	137.8 (4.3)	114.4 (6.1)	157.6 (5.2)
Serum HDL (mg/dl)	47.3 (0.3)	50.9 (0.5)	46.2 (0.4)
Serum glucose (mg/dl)	108.5 (1.1)	106.4 (1.5)	109.9 (1.5)
	**N** (**%**) **^b^**		
Metabolic syndrome ^c^	607 (21.8)	236 (18.0)	304 (18.9)
Education			
<High school	464 (12.9)	294 (22.9)	591 (42.9)
High school graduate	603 (23.4)	306 (26.5)	269 (21.3)
College or higher	1316 (59.9)	500 (44.9)	380 (30.9)
Poverty to income ratio (PIR)			
PIR < 1.0	332 (7.8)	243 (19.7)	340 (25.2)
PIR ≥ 1.0	2013 (86.6)	868 (71.6)	849 (64.3)
Smoking status			
Current smoker	582 (22.5)	322 (28.3)	281 (22.1)
Former smoker	862 (30.6)	252 (16.7)	335 (20.6)
Never smoker	961 (43.8)	540 (50.2)	635 (52.8)
Fasted ≥ 8 h ^d^			
Yes	1107 (44.4)	468 (38.6)	615 (45.2)
No	112 (3.9)	85 (7.1)	56 (4.1)

^a^ Values are reported as weighted sample mean and standard error of the mean (SE). ^b^ Weighted percentages accounting for sampling design. ^c^ *p* = 0.049, ^d^ *p* = 0.006. All other comparisons were significant at *p* < 0.0001. *p* values are generated from *t*-tests (continuous variables) and chi square tests (categorical variables). Total percentages do not equal 100 as missing values were included in the denominator.

**Table 2:** Demographic and metabolic characteristics among women, NHANES (2005–2014).

	White (n = 2367)	Black (n = 1200)	Mexican/Hispanic (n = 1390)
	Mean (SE) ^a^		
Age (years)	49.2 (0.4)	44.2 (0.5)	41.1 (0.4)
Body mass index (kg/m^2^)	28.2 (0.2)	31.7 (0.3)	29.3 (0.2)
Total calorie intake (Kcal)	1824 (16.9)	1863 (30.2)	1791 (25.1)
Urinary creatinine (mg/dl)	96.7 (1.6)	150.7 (2.6)	106.4 (2.1)
Waist circumference (cm)	94.9 (0.4)	100.2 (0.6)	95.4 (0.5)
Systolic blood pressure (mm Hg)	120.3 (0.4)	122.5 (0.6)	116.6 (0.5)
Diastolic blood pressure (mm Hg)	68.7 (0.3)	69.9 (0.4)	67.4 (0.3)
Serum triglycerides (mg/dl)	121.4 (2.9)	91.0 (2.7)	120.0 (3.2)
Serum HDL (mg/dl)	58.8 (0.4)	58.4 (0.5)	53.1 (0.4)
Serum glucose (mg/dl)	102.7 (0.8)	105.7 (1.9)	107.2 (2.1)
	**N** (**%**) **^b^**		
Metabolic syndrome ^c^	595 (22.2)	316 (23.6)	345 (18.4)
Education			
<High school	373 (11.7)	289 (21.7)	608 (41.1)
High school graduate	604 (23.9)	265 (22.8)	249 (19.3)
College or higher	1296 (61.2)	555 (50.9)	434 (34.8)
Poverty to income ratio (PIR)			
PIR < 1.0	416 (11.2)	297 (25.0)	417 (29.7)
PIR ≥ 1.0	1802 (82.7)	801 (67.2)	794 (59.1)
Smoking status			
Current smoker	548 (20.9)	218 (18.8)	154 (12.4)
Former smoker	562 (24.4)	170 (12.6)	204 (12.9)
Never smoker	1183 (52.3)	740 (65.1)	950 (71.0)
Menopause status			
Yes	1234 (47.9)	565 (39.8)	577 (28.9)
No	955 (44.7)	507 (48.7)	675 (59.6)
Fasted ≥ 8 h ^d^			
Yes	1067 (44.4)	491 (41.0)	611 (42.7)
No	68 (2.6)	51 (4.4)	43 (2.9)

^a^ Values are reported as weighted sample mean and standard error of the mean (SE). ^b^ Weighted percentages account for sampling design. ^c^ *p* = 0.059, ^d^ *p* = 0.078. All other comparisons were significant at *p* < 0.0001. *p* values generated from *t*-tests (continuous variables) and chi square tests (categorical variables). Total percentages do not equal 100 as missing values were included in the denominator.

**Table 3:** Urinary phthalate metabolites among men and women by race, NHANES (2005–2014).

Phthalate Metabolites (ng/mL)	Men			Women		
	White (n = 2503)	Black (n = 1211)	Mexican/Hispanic (n = 1346)	White (n= 2367)	Black (n = 1200)	Mexican/Hispanic (n = 1390)
	Geometric Mean (95% CI)			Geometric Mean (95% CI)		
**∑ LMW**	94.0 (87.4, 101.1)	199.5 (175.5, 226.9)	154.0 (137.6, 172.2)	86.8 (80.3, 93.8)	214.5 (192.8, 238.7)	155.3 (135.2, 178.5)
MEP	59.4 (54.9, 64.2)	145.2 (123.8, 170.3)	107.6 (95.4, 121.3)	56.6 (51.8, 61.9)	153.7 (135.4, 174.4)	106.0 (90.9, 123.7)
MiBP	5.8 (5.5, 6.2)	9.3 (8.4, 10.4)	8.5 (7.7, 9.3)	4.7 (4.4, 5.0)	10.7 (9.8, 11.7)	8.4 (7.6, 9.2)
MnBP	12.2 (11.2, 13.1)	16.5 (15.0, 18.2)	14.5 (13.1, 16.1)	11.1 (10.2, 12.0)	20.6 (18.7, 22.6)	16.0 (14.2, 18.1)
**∑** **HMW**	94.9 (87.7, 102.8)	100.7 (92.4, 109.7)	97.8 (87.8, 109.0)	72.7 (66.9, 79.1)	104.6 (96.6, 113.4)	82.0 (75.3, 89.2)
MCNP	2.9 (2.7, 3.1)	3.0 (2.8, 3.3)	2.5 (2.3, 2.7)	2.1 (1.9, 2.3)	2.9 (2.6, 3.1)	2.1 (1.9, 2.3)
MCOP	11.6 (10.3, 13.1)	11.1 (9.7, 12.6)	11.1 (9.7, 12.5)	8.5 (7.7, 9.5)	11.6 (10.0, 13.4)	9.7 (8.5, 11.2)
MCPP	2.7 (2.5, 3.0)	2.8 (2.5, 3.1)	2.5 (2.2, 2.8)	2.0 (1.9, 2.2)	2.6 (2.3, 2.9)	2.1 (1.8, 2.3)
MBzP	5.8 (5.3, 6.2)	7.3 (6.6, 8.0)	5.8 (5.2, 6.5)	4.7 (4.3, 5.1)	7.9 (7.1, 8.8)	5.1 (4.5, 5.7)
**∑** **DEHP**	49.2 (44.8, 54.0)	53.4 (47.1, 60.6)	53.2 (46.4, 61.0)	38.0 (34.5, 41.9)	55.7 (50.0, 61.9)	46.0 (41.9, 50.5)
MECPP	22.3 (20.3, 24.5)	22.2 (19.5, 25.2)	25.0 (21.8, 28.6)	17.6 (15.9, 19.4)	23.9 (21.5, 26.6)	21.7 (19.7, 23.9)
MEHHP	14.8 (13.4, 16.3)	17.0 (14.9, 19.4)	15.5 (13.4, 17.9)	11.0 (9.9, 12.1)	17.0 (15.1, 19.0)	13.0 (11.7, 14.3)
MEHP	1.7 (1.6, 1.9)	2.4 (2.1, 2.7)	2.3 (2.0, 2.6)	1.3 (1.2,1.4)	2.2 (2.0, 2.5)	1.8 (1.7, 2.0)
MEOHP	8.6 (7.8, 9.5)	9.8 (8.6, 11.1)	8.9 (7.8, 10.2)	6.7 (6.0, 7.4)	10.6 (9.5, 11.8)	8.1 (7.3, 8.9)

**Table 4:** Associations between urinary phthalate metabolite concentrations and metabolic syndrome among men by race, NHANES (2005–2014).

Phthalate Metabolites	White (n = 2503)		Black (n = 1211)		Mexican/Hispanic (n = 1346)	
	n	Multivariable ^a^ OR (95% CI)	n	Multivariable ^a^ OR (95% CI)	n	Multivariable ^a^ OR (95% CI)
**MCNP**						
Q1	514	1.00	230	1.00	293	1.00
Q2	610	0.88 (0.56, 1.38)	298	1.39 (0.75, 2.58)	365	0.94 (0.56, 1.56)
Q3	667	1.00 (0.62, 1.61)	302	0.92 (0.46, 1.86)	362	0.80 (0.45, 1.44)
Q4	712	0.75 (0.49, 1.16)	381	0.80 (0.42, 1.52)	326	1.06 (0.59, 1.92)
*p* for trend		0.29		0.20		0.92
**MCOP**						
Q1	626	1.00	283	1.00	303	1.00
Q2	585	1.16 (0.76, 1.76)	289	1.14 (0.67, 1.95)	387	1.28 (0.76, 2.16)
Q3	669	0.87 (0.54, 1.39)	288	1.94 (1.16, 3.25)	324	1.10 (0.58, 2.07)
Q4	623	1.02 (0.69, 1.52)	351	1.19 (0.68, 2.06)	332	0.86 (0.49, 1.50)
*p* for trend		0.71		0.28		0.40
**MECPP**						
Q1	572	1.00	278	1.00	247	1.00
Q2	632	0.87 (0.59, 1.28)	334	1.20 (0.73, 1.96)	338	0.67 (0.38, 1.19)
Q3	612	1.12 (0.76, 1.65)	298	0.71 (0.39, 1.28)	383	1.11 (0.60, 2.06)
Q4	687	1.38 (0.88, 2.16)	301	1.45 (0.75, 2.79)	378	1.23 (0.65, 2.30)
*p* for trend		0.06		0.53		0.19
**MnBP**						
Q1	660	1.00	233	1.00	283	1.00
Q2	709	1.29 (0.94, 1.77)	298	0.64 (0.36, 1.12)	350	1.32 (0.86, 2.05)
Q3	634	0.95 (0.65, 1.40)	352	0.71 (0.44, 1.14)	347	1.30 (0.78, 2.15)
Q4	500	0.93 (0.59, 1.46)	328	0.71 (0.39,1.28)	366	1.89 (0.97, 3.65)
*p* for trend		0.44		0.34		0.10
**MCPP**						
Q1	552	1.00	254	1.00	326	1.00
Q2	559	0.81 (0.55, 1.18)	297	1.14 (0.65, 1.20)	337	1.09 (0.72, 1.64)
Q3	687	0.88 (0.60, 1.29)	332	1.42 (0.85, 2.37)	364	0.91 (0.57, 1.47)
Q4	705	0.97 (0.62, 1.51)	328	1.31 (0.79, 2.16)	319	1.19 (0.72, 1.98)
*p* for trend		0.91		0.17		0.68
**MEP**						
Q1	768	1.00	157	1.00	229	1.00
Q2	697	0.85 (0.57, 1.26)	278	0.61 (0.35, 1.05)	325	1.02 (0.50, 2.08)
Q3	562	0.79 (0.56, 1.11)	344	0.61 (0.33, 1.14)	371	1.11 (0.55, 2.26)
Q4	476	0.95 (0.64, 1.41)	432	0.43 (0.24, 0.77)	421	1.04 (0.52, 2.08)
*p* for trend		0.66		0.01		0.86
**MEHHP**						
Q1	561	1.00	228	1.00	262	1.00
Q2	634	1.01 (0.69, 1.48)	303	1.25 (0.72, 2.18)	350	1.24 (0.66, 2.33)
Q3	657	0.85 (0.57, 1.27)	347	1.14 (0.62, 2.11)	353	1.51 (0.81, 2.81)
Q4	651	1.29 (0.90, 1.84)	333	1.57 (0.90, 2.73)	381	1.57 (0.84, 2.96)
*p* for trend		0.21		0.19		0.12
**MEHP**						
Q1	428	1.00	155	1.00	183	1.00
Q2	882	0.85 (0.60, 1.19)	339	1.04 (0.57, 1.90)	395	0.69 (0.41, 1.17)
Q3	626	0.93 (0.61, 1.41)	320	1.26 (0.77, 2.07)	359	0.67 (0.35, 1.27)
Q4	567	0.93 (0.59, 1.44)	397	0.93 (0.51, 1.68)	409	0.65 (0.32, 1.33)
*p* for trend		0.93		0.87		0.31
**MiBP**						
Q1	732	1.00	197	1.00	225	1.00
Q2	731	0.81 (0.57, 1.16)	268	0.92 (0.50, 1.70)	351	1.99 (1.00, 3.95)
Q3	594	0.73 (0.48, 1.10)	340	1.01 (0.60, 1.69)	391	2.13 (1.10, 4.13)
Q4	446	0.91 (0.56, 1.48)	406	1.61 (0.83, 3.12)	379	1.87 (0.80, 4.36)
*p* for trend		0.59		0.08		0.30
**MEOHP**						
Q1	567	1.00	233	1.00	275	1.00
Q2	645	1.18 (0.80, 1.73)	311	1.68 (0.99, 2.83)	362	0.88 (0.47, 1.64)
Q3	654	0.97 (0.67, 1.40)	331	1.24 (0.65, 2.36)	343	1.18 (0.63, 2.20)
Q4	637	1.42 (0.96, 2.10)	336	1.62 (0.96, 2.73)	366	1.38 (0.71, 2.70)
*p* for trend		0.12		0.28		0.17
**MBzP**						
Q1	560	1.00	208	1.00	320	1.00
Q2	654	1.45 (1.05, 2.01)	308	0.73 (0.40, 1.32)	349	1.51 (0.91, 2.52)
Q3	658	1.03 (0.73, 1.44)	345	1.03 (0.65, 1.63)	356	1.16 (0.66, 2.03)
Q4	631	1.24 (0.89, 1.73)	350	0.66 (0.31, 1.39)	321	1.94 (0.95, 3.98)
*p* for trend		0.71		0.47		0.15

^a^ Adjusted for age, urinary creatinine, total caloric intake, education, smoking status, fasting status, and poverty level. Quartile values (ng/mL): MCNP: Q1 = 0.1- 1.2, Q2 = 1.2- 2.4, Q3 = 2.4–4.7, Q4 = 4.7–730.3; MCOP: Q1 = 0.1–3.7, Q2 = 3.7–8.4, Q3 = 8.4–23.2, Q4 = 23.3–3287.4; MECPP: Q1 = 0.1–9.6, Q2 = 9.7–20.1, Q3 = 20.2–43.2, Q4 = 43.4–15,828.0; MnBP: Q1 = 0.2–7.1, Q2 = 7.1–15.4, Q3 = 15.4–31.4, Q4 = 31.4–25,863.0; MCPP: Q1 = 0.1–1.1, Q2 = 1.1–2.4, Q3 = 2.4–4.9, Q4 = 4.9–1588.7; MEP: Q1 = 0.3–26.9, Q2 = 26.9–74.6, Q3 = 74.6–240.7, Q4 = 240.8–31,660.0; MEHHP: Q1 = 0.1–5.8, Q2 = 5.8–13.1, Q3 = 13.1–29.3, Q4 = 29.3–9326.1; MEHP: Q1 = 0.3–0.6, Q2 = 0.6–1.7, Q3 = 1.7–4.1, Q4 = 4.1–1966.1; MiBP: Q1 = 0.1–3.5, Q2 = 3.5–7.7, Q3 = 7.7–15.0, Q4 = 15.1–14,537.0; MEOHP: Q1 = 0.1–3.6, Q2 = 3.6–8.0, Q3 = 8.0–17.4, Q4 = 17.4–6079.9; MBzP: Q1 = 0.1–2.5, Q2 = 2.5–6.0, Q3 = 6.0–13.9, Q4 = 13.9–16,510.3.

**Table 5:** Associations between urinary phthalate metabolite concentrations and metabolic syndrome among women by race, NHANES (2005–2014).

Phthalate Metabolites	White (n = 2367)		Black (n = 1200)		Mexican/Hispanic (n = 1390)	
	n	Multivariable ^a^ OR (95% CI)	n	Multivariable ^a^ OR (95% CI)	n	Multivariable ^a^ OR (95% CI)
**MCNP**						
Q1	745	1.00	268	1.00	395	1.00
Q2	579	1.08 (0.78, 1.49)	284	1.53 (0.96, 2.44)	367	1.57 (0.97, 2.55)
Q3	521	1.00 (0.67, 1.48)	311	1.24 (0.70, 2.18)	371	1.11 (0.64, 1.94)
Q4	522	1.07 (0.70, 1.64)	337	0.96 (0.61, 1.49)	257	1.76 (0.88, 3.50)
*p* for trend		0.86		0.51		0.27
**MCOP**						
Q1	680	1.00	249	1.00	355	1.00
Q2	595	1.14 (0.78, 1.66)	291	1.07 (0.64, 1.78)	378	1.46 (0.94, 2.30)
Q3	547	1.28 (0.80, 2.05)	329	1.10 (0.65, 1.84)	364	1.17 (0.64, 2.15)
Q4	545	1.68 (1.24, 2.29)	331	0.98 (0.60, 1.62)	293	1.09 (0.64, 1.86)
*p* for trend		0.001		0.93		0.92
**MECPP**						
Q1	759	1.00	262	1.00	283	1.00
Q2	549	1.02 (0.77, 1.35)	276	1.15 (0.70, 1.89)	340	1.39 (0.76, 2.56)
Q3	543	1.10 (0.79, 1.55)	321	0.50 (0.27, 0.93)	397	1.36 (0.63, 2.90)
Q4	516	1.08 (0.77, 1.51)	341	0.79 (0.46, 1.34)	370	1.11 (0.55, 2.22)
*p* for trend		0.61		0.11		0.86
**MnBP**						
Q1	773	1.00	193	1.00	285	1.00
Q2	552	0.83 (0.60, 1.15)	267	0.88 (0.52, 1.47)	335	1.42 (0.88, 2.29)
Q3	529	1.07 (0.73, 1.57)	296	0.89 (0.49, 1.61)	357	1.14 (0.55, 2.34)
Q4	513	0.81 (0.51, 1.29)	444	0.57 (0.30, 1.08)	413	1.01 (0.48, 2.14)
*p* for trend		0.60		0.10		0.79
**MCPP**						
Q1	749	1.00	282	1.00	399	1.00
Q2	532	0.83 (0.61, 1.12)	278	0.89 (0.55, 1.45)	365	1.10 (0.68, 1.77)
Q3	519	1.12 (0.77, 1.62)	317	0.84 (0.54, 1.31)	323	1.63 (0.80, 3.33)
Q4	567	1.37 (0.97, 1.94)	323	0.78 (0.42, 1.44)	303	0.87 (0.49, 1.54)
*p* for trend		0.05		0.41		0.99
**MEP**						
Q1	791	1.00	133	1.00	253	1.00
Q2	619	1.12 (0.82, 1.53)	272	1.33 (0.61, 2.90)	320	1.12 (0.68, 1.84)
Q3	542	1.09 (0.77, 1.53)	368	0.95 (0.39, 2.27)	384	1.32 (0.78, 2.22)
Q4	415	0.93 (0.63, 1.38)	427	0.75 (0.33, 1.74)	433	0.89 (0.46, 1.72)
*p* for trend		0.80		0.11		0.75
**MEHHP**						
Q1	781	1.00	235	1.00	307	1.00
Q2	565	0.89 (0.66, 1.20)	271	1.30 (0.71, 2.40)	364	1.14 (0.64, 2.03)
Q3	509	1.17 (0.85, 1.60)	313	0.88 (0.48, 1.60)	380	1.37 (0.68, 2.78)
Q4	512	0.86 (0.57, 1.32)	381	0.82 (0.42, 1.60)	339	1.02 (0.56, 1.87)
*p* for trend		0.76		0.26		0.79
**MEHP**						
Q1	535	1.00	185	1.00	210	1.00
Q2	939	0.82 (0.58, 1.17)	359	0.81 (0.49, 1.36)	467	0.84 (0.46, 1.54)
Q3	486	0.73 (0.50, 1.05)	277	0.80 (0.45, 1.41)	353	0.90 (0.46, 1.78)
Q4	407	0.53 (0.35, 0.80)	379	0.52 (0.27, 1.02)	360	0.64 (0.31, 1.30)
*p* for trend		0.003		0.07		0.26
**MiBP**						
Q1	847	1.00	201	1.00	273	1.00
Q2	635	1.07 (0.76, 1.52)	230	0.62 (0.37, 1.04)	328	2.16 (1.37, 3.41)
Q3	486	1.09 (0.74, 1.61)	298	0.90 (0.47, 1.72)	391	1.56 (0.87, 2.80)
Q4	399	1.17 (0.71, 1.92)	471	1.03 (0.59, 1.79)	398	1.70 (0.92, 3.13)
*p* for trend		0.53		0.49		0.36
**MEOHP**						
Q1	767	1.00	236	1.00	302	1.00
Q2	562	0.76 (0.58, 0.99)	255	1.47 (0.75, 2.87)	356	1.41 (0.84, 2.35)
Q3	526	1.00 (0.71, 1.40)	323	1.00 (0.54, 1.83)	378	1.28 (0.65, 2.54)
Q4	512	0.78 (0.52, 1.16)	386	0.87 (0.43, 1.74)	354	1.26 (0.62, 2.56)
*p* for trend		0.43		0.33		0.64
**MBzP**						
Q1	694	1.00	223	1.00	383	1.00
Q2	563	0.84 (0.59, 1.22)	255	1.08 (0.63, 1.85)	370	0.70 (0.43, 1.15)
Q3	535	1.14 (0.78, 1.68)	327	0.90 (0.52, 1.56)	332	0.99 (0.55, 1.77)
Q4	575	1.28 (0.80, 2.05)	395	1.12 (0.61, 2.05)	305	0.67 (0.34, 1.30)
*p* for trend		0.19		0.88		0.42

^a^ Adjusted for age, urinary creatinine, total caloric intake, education, smoking status, fasting status, and poverty level. Quartile values (ng/mL): MCNP: Q1 = 0.1–1.2, Q2 = 1.2–2.4, Q3 = 2.4–4.7, Q4 = 4.7–730.3; MCOP: Q1 = 0.1–3.7, Q2 = 3.7–8.4, Q3 = 8.4–23.2, Q4 = 23.3–3287.4; MECPP: Q1 = 0.1–9.6, Q2 = 9.7–20.1, Q3 = 20.2–43.2, Q4 = 43.4–15,828.0; MnBP: Q1 = 0.2–7.1, Q2 = 7.1–15.4, Q3 = 15.4–31.4, Q4 = 31.4–25,863.0; MCPP: Q1 = 0.1–1.1, Q2 = 1.1–2.4, Q3 = 2.4–4.9, Q4 = 4.9–1588.7; MEP: Q1 = 0.3–26.9, Q2 = 26.9–74.6, Q3 = 74.6–240.7, Q4 = 240.8–31,660.0; MEHHP: Q1 = 0.1–5.8, Q2 = 5.8–13.1, Q3 = 13.1–29.3, Q4 = 29.3–9326.1; MEHP: Q1 = 0.3–0.6, Q2 = 0.6–1.7, Q3 = 1.7–4.1, Q4 = 4.1–1966.1; MiBP: Q1 = 0.1–3.5, Q2 = 3.5–7.7, Q3 = 7.7–15.0, Q4 = 15.1–14,537.0; MEOHP: Q1 = 0.1–3.6, Q2 = 3.6–8.0, Q3 = 8.0–17.4, Q4 = 17.4–6079.9; MBzP: Q1 = 0.1–2.5, Q2 = 2.5–6.0, Q3 = 6.0–13.9, Q4 = 13.9–16,510.3.

**Table 6:** Associations between urinary phthalate metabolite summary measures and metabolic syndrome among men by race, NHANES (2005–2014).

Phthalate Metabolites	White (n = 2503)		Black (n = 1211)		Mexican/Hispanic (n = 1346)	
	n	Multivariable ^a^ OR (95% CI)	n	Multivariable ^a^ OR (95% CI)	n	Multivariable ^a^ OR (95% CI)
**∑ LMW**						
Q1	746	1.00	166	1.00	214	1.00
Q2	704	0.81 (0.53, 1.22)	275	0.48 (0.26, 0.87)	340	0.77 (0.39, 1.52)
Q3	573	0.79 (0.51, 1.21)	353	0.46 (0.26, 0.81)	377	1.10 (0.54, 2.25)
Q4	480	0.93 (0.60, 1.44)	417	0.42 (0.24, 0.75)	415	0.90 (0.47, 1.73)
*p* for trend		0.70		0.02		0.90
**∑ HMW**						
Q1	559	1.00	226	1.00	271	1.00
Q2	633	0.77 (0.54, 1.09)	324	1.36 (0.75, 2.46)	363	0.90 (0.51, 1.58)
Q3	652	1.04 (0.72, 1.50)	326	1.41 (0.76, 2.63)	337	1.04 (0.54, 2.00)
Q4	659	1.11 (0.72, 1.71)	335	1.18 (0.62, 2.23)	375	1.15 (0.60, 2.18)
*p* for trend		0.26		0.70		0.50
**∑ DEHP**						
Q1	575	1.00	247	1.00	246	1.00
Q2	621	1.22 (0.84, 1.77)	317	1.33 (0.78, 2.28)	360	0.99 (0.57, 1.73)
Q3	651	1.11 (0.78, 1.59)	323	1.01 (0.59, 1.72)	361	1.12 (0.59, 2.14)
Q4	656	1.54 (1.01, 2.35)	324	1.49 (0.91, 2.43)	379	1.40 (0.75, 2.64)
*p* for trend		0.06		0.32		0.21

^a^ Adjusted for age, urinary creatinine, total caloric intake, education, smoking status, fasting status, and poverty level. ∑ LMW: MEP, MnBP, and MiBP. ∑ HMW: MCNP, MCOP, MCPP, MBzP, and DEHP (MEHP, MECPP, MEHHP, and MEOHP). ∑ DEHP: MEHP, MECPP, MEHHP, and MEOHP. Quartile values (ng/mL): ∑ LMW: Q1 = 0.7–48.3, Q2 = 48.3–116.8, Q3 = 116.8–298.5, Q4 = 298.6–31,764.6; ∑ HMW: Q1 = 1.6–40.6, Q2 = 40.6–83.4, Q3 = 83.4–168.8, Q4 = 169.0–32,066.2; ∑ DEHP: Q1 = 0.6–20.6, Q2 = 20.7–44.3, Q3 = 44.3–94.6, Q4 = 94.7–32,041.5.

**Table 7:** Associations between urinary phthalate metabolite summary measures and metabolic syndrome among women by race, NHANES (2005–2014).

Phthalate Metabolites	White (n = 2367)		Black (n = 1200)		Mexican/Hispanic (n = 1390)	
	n	Multivariable ^a^ OR (95% CI)	n	Multivariable ^a^ OR (95% CI)	n	Multivariable ^a^ OR (95% CI)
**∑ LMW**						
Q1	839	1.00	141	1.00	242	1.00
Q2	595	1.10 (0.83, 1.47)	244	0.97 (0.44, 2.14)	339	1.17 (0.70, 1.93)
Q3	522	0.96 (0.64, 1.45)	379	0.97 (0.49, 1.92)	369	1.28 (0.77, 2.15)
Q4	411	0.84 (0.58, 1.21)	436	0.63 (0.30, 1.34)	440	0.85 (0.48, 1.50)
*p* for trend		0.34		0.12		0.54
**∑ HMW**						
Q1	766	1.00	236	1.00	355	1.00
Q2	540	1.11 (0.81, 1.51)	279	1.39 (0.85, 2.26)	346	1.71 (1.04, 2.79)
Q3	549	1.46 (1.05, 2.02)	316	0.84 (0.42, 1.71)	374	1.24 (0.64, 2.39)
Q4	512	1.15 (0.83, 1.57)	369	0.85 (0.46, 1.59)	315	1.12 (0.62, 2.03)
*p* for trend		0.15		0.29		0.95
**∑ DEHP**						
Q1	782	1.00	242	1.00	288	1.00
Q2	558	0.90 (0.69, 1.16)	265	1.38 (0.76, 2.50)	363	1.17 (0.70, 1.99)
Q3	514	1.14 (0.80, 1.63)	318	0.68 (0.37, 1.23)	396	1.24 (0.61, 2.53)
Q4	513	0.93 (0.62, 1.41)	375	0.79 (0.43, 1.47)	343	0.94 (0.49, 1.78)
*p* for trend		0.99		0.14		0.87

^a^ Adjusted for age, urinary creatinine, total caloric intake, education, smoking status, fasting status, and poverty level. ∑ LMW: MEP, MnBP, and MiBP. ∑ HMW: MCNP, MCOP, MCPP, MBzP, and DEHP (MEHP, MECPP, MEHHP, and MEOHP). ∑ DEHP: MEHP, MECPP, MEHHP, and MEOHP. Quartile values (ng/mL): ∑ LMW: Q1 = 0.7–48.3, Q2 = 48.3–116.8, Q3 = 116.8–298.5, Q4 = 298.6–31,764.6; ∑ HMW: Q1 = 1.6–40.6, Q2 = 40.6–83.4, Q3 = 83.4–168.8, Q4 = 169.0–32,066.2; ∑ DEHP: Q1 = 0.6–20.6, Q2 = 20.7–44.3, Q3 = 44.3–94.6, Q4 = 94.7–32,041.5.

## Data Availability

This analysis utilizes publicly available data, which can be downloaded from https://wwwn.cdc.gov/nchs/nhanes/, accessed on 18 June 2021.

## Supplementary Materials

The following are enclosed, available online at https://www.mdpi.com/article/10.3390/ijerph18136870/s1. Table S1: Urinary Phthalate Metabolite Distributions, NHANES (2005–2014), Table S2: Demographic and Metabolic Characteristics among Men with and without Metabolic Sydnrome (MetS), NHANES (2005–2014), Table S3: Demographic and Metabolic Characteristics among Women with and without Metabolic Sydnrome (MetS), NHANES (2005–2014), Table S4: Urinary Phthalate Metabolites Stratified by Sex, NHANES (2005–2014), Table S5: Spearman Correlation Coefficients between Urinary Phthalate Metabolites, NHANES (2005–2014), Table S6: Overall Associations between Urinary Phthalate Metabolites and Metabolic Syndrome, NHANES (2005–2014), Table S7: Associations between Urinary Phthalate Metabolites and Metabolic Syndrome by Sex, NHANES (2005–2014), Table S8: Overall Associations between Urinary Phthalate Metabolite Summary Measures and Metabolic Syndrome, NHANES (2005–2014), and Table S9: Associations between Urinary Phthalate Metabolite Summary Measures and Metabolic Syndrome by Sex, NHANES (2005–2014).
