# Supplementary material for: Racial and Sex Differences between Urinary Phthalates and Metabolic Syndrome among U.S. Adults: NHANES 2005–2014
Source: Int J Environ Res Public Health. 2021 Jun 26;18(13):6870. doi: 10.3390/ijerph18136870 (PMC8297378; doi:10.3390/ijerph18136870)
Supplement: Supplementary file 1 [file ijerph-18-06870-s001.zip › ijerph-1265115-supplementary.pdf]

International Journal of
Environmental Research and Public Health

MDPI

Racial and Sex Differences between Urinary Phthalates and Metabolic Syndrome among U.S. Adults: NHANES 2005-2014

Rajrupa Ghosh [1], Mefruz Haque [1], Paul C. Turner [2], Raul Cruz-Cano [1], Cher M. Dallal [1,*]

Department of Epidemiology and Biostatistics, School of Public Health, University of Maryland, College Park, Maryland, 20742, USA; rajrupa@terpmail.umd.edu (R.G.); haque.mefruz@gmail.com (M.H.); raulcruz@umd.edu (R.C.-C.)

[2] Maryland Institute for Applied Environmental Health, School of Public Health, University of Maryland, College Park, Maryland, 20742, USA; pturner3@umd.edu

* Correspondence: cdallal@umd.edu; Tel: 301-405-7065

Supplemental Tables

Table S1. Urinary Phthalate Metabolite Distributions, NHANES (2005-2014).

Phthalate Metabolites	LOD* ng/ml	% < LOD Reported by NHANES**	% < LOD Analytic File***	Geometric Mean (95%CI) ng/ml	Range ng/ml
**∑ LMW**	-	-	-	108.0 (102.4, 113.8)	0.7 to 31764
**MEP**	0.5 – 1.2	(0.1)	(0.1)	71.1 (67.1, 75.3)	0.2 to 31660
**MiBP**	0.2 – 0.8	(1.9)	(4.5)	6.1 (5.8, 6.4)	0.1 to 14537
**MnBP**	0.4 – 0.6	(1.6)	(1.8)	12.8 (12.1, 13.5)	0.2 to 25863
**∑ HMW**	-	-	-	86.1 (81.2, 91.5)	1.6 to 32066
**MCNP**	0.2 – 0.6	(5.8)	(8.8)	2.5 (2.4, 2.7)	0.1 to 730
**MCOP**	0.2 – 0.7	(2.3)	(2.6)	10.1 (9.4, 11.0)	0.1 to 3287
**MCPP**	0.2 – 0.4	(4.0)	(6.8)	2.4 (2.2, 2.5)	0.1 to 1589
**MBzP**	0.2 – 0.3	(1.8)	(2.3)	5.5 (5.2, 5.8)	0.1 to 16510
**∑ DEHP**	-	-	-	45.3 (42.3, 48.6)	0.6 to 32042
**MECPP**	0.2 – 0.6	(0.2)	(0.2)	20.6 (19.3, 22.1)	0.1 to 15828
**MEHHP**	0.2 – 0.7	(0.6)	(1.2)	13.4 (12.5, 14.4)	0.1 to 9326
**MEHP**	0.5 – 1.2	(31.6)	(40.0)	1.7 (1.6, 1.8)	0.2 to 1966
**MEOHP**	0.2 – 0.7	(1.2)	(2.5)	8.0 (7.4, 8.6)	0.1 to 6080

*LOD: Range of LODs across NHANES survey years 2005-2014. **% < LOD: Reported by NHANES for each survey year was used to determine the % < LOD across survey years 2005-2014. ***% < LOD: The highest LOD across the survey years 2005-2014 was used to determine the % of metabolites below LOD in the analytic file.

Table S2. Demographic and Metabolic Characteristics among Men with and without Metabolic Sydnrome (MetS), NHANES (2005-2014).

	White (n=2,503)		Black (n=1,211)		Mexican/Hispanic (n=1,346)	
	**MetS** (n=607)	**No MetS** (n= 1896)	**MetS** (n= 236)	**No MetS** (n= 975)	**MetS** (n= 304)	**No MetS** (n= 1042)
			**Mean (SE)**[a]			
Age (years)	54.7 (0.7)	44.7 (0.4)	50.4 (1.1)	40.6 (0.5)	47.4 (0.9)	37.0 (0.4)
Body Mass Index (kg/m^2)	33.6 (0.3)	27.4 (0.1)	35.0 (0.5)	28.0 (0.2)	33.9 (0.4)	28.0 (0.2)
Total Calorie Intake (Kcal)	2418.6 (57.1)	2680.3 (28.9)	2264.4 (96.3)	2558.0 (42.3)	2566.6 (83.2)	2559.9 (42.1)
Urinary creatinine (mg/dl)	138.8 (3.6)	136.3 (2.1)	157.4 (6.6)	189.8 (3.4)	141.3 (4.2)	145.2 (2.7)
Waist Circumference (cm)	116.9 (0.6)	98.3 (0.4)	116.1 (1.1)	95.0 (0.6)	113.5 (1.0)	96.6 (0.5)
Systolic blood pressure (mm Hg)	131.1 (0.9)	121.8 (0.4)	136.0 (1.5)	125.3 (0.6)	131.5 (1.0)	119.2 (0.4)
Diastolic blood pressure (mm Hg)	73.9 (0.7)	71.2 (0.3)	75.9 (1.2)	72.2 (0.5)	75.9 (1.1)	68.6 (0.4)
Serum triglycerides (mg/dl)	206.1 (10.7)	110.5 (3.5)	164.9 (11.3)	101.0 (7.0)	241.3 (13.6)	128.3 (4.2)
Serum HDL (mg/dl)	38.4 (0.4)	49.9 (0.4)	41.3 (0.9)	52.9 (0.5)	37.9 (0.6)	48.2 (0.5)
Serum glucose (mg/dl)	128.6 (3.1)	100.6 (0.6)	129.5 (4.7)	100.1 (1.2)	128.3 (3.8)	103.4 (1.3)
			**N (%)**[b]			
Education						
< High school	152 (17.8)	312 (11.5)	70 (27.7)	224 (21.9)	142 (47.0)	449 (42.0)
High school graduate	155 (25.9)	448 (22.8)	67 (28.8)	239 (26.0)	65 (23.4)	204 (20.8)
College or higher	297 (55.9)	1019 (61.0)	90 (40.6)	410 (45.9)	93 (28.5)	287 (31.5)
Poverty to income ratio (PIR)						
PIR < 1.0	83 (8.9)	249 (7.5)	44 (17.7)	199 (20.1)	70 (22.5)	270 (25.8)
PIR ≥ 1.0	492 (86.6)	1521 (86.6)	179 (75.9)	689 (70.7)	200 (67.4)	649 (63.6)
Smoking status						
Current smoker	117 (18.1)	465 (23.7)	62 (31.3)	260 (27.6)	61 (23.5)	220 (21.8)
Former smoker	263 (38.8)	599 (28.4)	77 (24.0)	175 (15.1)	105 (28.3)	230 (18.8)
Never smoker	224 (42.7)	737 (44.1)	89 (42.0)	451 (51.9)	134 (47.1)	501 (54.2)
Fasted ≥ 8 hours						
Yes	345 (57.3)	762 (40.9)	107 (45.1)	361 (37.1)	178 (59.1)	437 (41.9)
No	37 (5.6)	75 (3.4)	23 (10.1)	62 (6.5)	19 (7.8)	37 (3.2)

[a]Values are reported as weighted sample mean and standard error of the mean (SE). [b]Weighted percentages accounting for sampling design.

Total percentages do not equal 100 as missing values were included in the denominator.

Table S3. Demographic and Metabolic Characteristics among Women with and without Metabolic Sydnrome (MetS), NHANES (2005-2014).

	White (n=2,367)		Black (n=1,200)		Mexican/Hispanic (n=1,390)	
	**MetS** (n=595)	**No MetS** (n= 1772)	**MetS** (n= 316)	**No MetS** (n= 884)	**MetS** (n= 345)	**No MetS** (n= 1045)
			**Mean (SE)**[a]			
Age (years)	58.1 (0.7)	46.8 (0.4)	54.7 (0.8)	41.0 (0.5)	53.9 (1.0)	38.2 (0.4)
Body Mass Index (kg/m^2)	33.8 (0.4)	26.7 (0.2)	35.6 (0.5)	30.5 (0.3)	33.5 (0.4)	28.3 (0.2)
Total Calorie Intake (Kcal)	1773.1 (30.6)	1838.8 (19.9)	1713.7 (48.9)	1908.7 (36.4)	1686.7 (62.9)	1815.1 (27.4)
Urinary creatinine (mg/dl)	101.0 (3.2)	95.4 (1.9)	139.0 (4.9)	154.4 (3.0)	104.1 (3.6)	107.0 (2.4)
Waist Circumference (cm)	109.1 (0.8)	90.7 (0.4)	111.4 (0.9)	96.7 (0.7)	106.0 (0.8)	92.9 (0.5)
Systolic blood pressure (mm Hg)	129.7 (0.8)	117.6 (0.4)	133.8 (1.2)	118.8 (0.6)	132.0 (1.4)	113.1 (0.5)
Diastolic blood pressure (mm Hg)	69.3 (0.6)	68.5 (0.3)	72.2 (0.9)	69.1 (0.4)	71.7 (0.8)	66.4 (0.4)
Serum triglycerides (mg/dl)	186.3 (6.8)	96.2 (2.3)	137.0 (7.0)	74.6 (1.9)	197.3 (7.6)	95.1 (2.1)
Serum HDL (mg/dl)	45.7 (0.5)	62.5 (0.5)	49.3 (0.8)	60.9 (0.6)	43.9 (0.6)	55.2 (0.5)
Serum glucose (mg/dl)	121.4 (2.2)	95.4 (0.6)	133.4 (5.1)	95.5 (1.4)	133.8 (6.5)	98.5 (1.4)

	White (n=2,367)		Black (n=1,200)		Mexican/Hispanic (n=1,390)	
			N (%)[b]			
Education						
< High school	129 (17.4)	244 (10.0)	102 (30.0)	187 (19.1)	212 (55.7)	396 (37.8)
High school graduate	196 (32.0)	408 (21.7)	82 (25.9)	183 (21.8)	49 (15.7)	200 (20.1)
College or higher	264 (50.0)	1032 (64.5)	130 (43.8)	425 (53.1)	84 (28.7)	350 (36.2)
Poverty to income ratio (PIR)						
PIR < 1.0	119 (12.6)	297 (10.8)	66 (21.4)	231 (26.2)	106 (30.4)	311 (29.6)
PIR ≥ 1.0	432 (80.8)	1370 (83.2)	214 (68.0)	587 (66.9)	187 (54.0)	607 (60.2)
Smoking status						
Current smoker	146 (22.9)	402 (20.3)	73 (24.9)	145 (16.9)	38 (13.2)	116 (12.2)
Former smoker	149 (27.7)	413 (23.4)	64 (17.8)	106 (11.0)	78 (21.1)	126 (11.0)
Never smoker	297 (49.1)	886 (53.1)	177 (57.0)	563 (67.6)	228 (65.5)	722 (72.2)
Menopause status						
Yes	436 (70.9)	798 (41.4)	238 (67.8)	327 (31.2)	251 (57.4)	326 (22.5)
No	130 (24.4)	825 (50.5)	57 (25.5)	450 (55.8)	66 (32.3)	609 (65.7)
Fasted ≥ 8 hours						
Yes	344 (57.1)	723 (40.8)	158 (50.0)	333 (38.2)	188 (56.2)	423 (39.6)
No	17 (2.8)	51 (2.5)	14 (4.0)	37 (4.5)	13 (3.7)	30 (2.7)

[a]Values are reported as weighted sample mean and standard error of the mean (SE). [b]Weighted percentages accounting for sampling design.

Total percentages do not equal 100 as missing values were included in the denominator.

Table S4. Urinary Phthalate Metabolites Stratified by Sex, NHANES (2005-2014).

Phthalate Metabolites (ng/ml)	Men (n=5,060)	Women (n=4,957)
	Geometric Mean (95% CI)	
∑ LMW	110.3 (104.3, 116.7)	105.8 (98.7, 113.3)
MEP	71.9 (67.4, 76.7)	70.3 (65.0, 76.0)
MiBP	6.5 (6.2, 6.9)	5.6 (5.3, 6.0)
MnBP	12.9 (12.1, 13.7)	12.6 (11.8, 13.5)
∑ HMW	96.0 (89.6, 102.9)	77.5 (72.6, 82.8)
MCNP	2.9 (2.7, 3.0)	2.2 (2.0, 2.4)
MCOP	11.5 (10.5, 12.6)	9.1 (8.3, 9.8)
MCPP	2.7 (2.5, 2.9)	2.1 (2.0, 2.2)
MBzP	5.9 (5.6, 6.3)	5.1 (4.7, 5.5)
∑ DEHP	50.2 (46.3, 54.5)	41.0 (38.0, 44.3)
MECPP	22.7 (20.9, 24.6)	18.8 (17.4, 20.3)
MEHHP	15.1 (13.9, 16.5)	11.9 (11.0, 12.9)
MEHP	1.9 (1.7, 2.1)	1.5 (1.4, 1.6)
MEOHP	8.8 (8.1, 9.6)	7.3 (6.7, 7.9)

Table S5. Spearman Correlation Coefficients between Urinary Phthalate Metabolites, NHANES (2005-2014).

	MCNP	MCOP	MECPP	MnBP	MCPP	MEP	MEHHP	MEHP	MiBP	MEOHP	MBzP
MCNP		0.60	0.46	0.38	0.61	0.21	0.41	0.35	0.41	0.43	0.36
MCOP			0.34	0.26	0.68	0.08	0.30	0.27	0.40	0.33	0.27
MECPP				0.61	0.56	0.36	0.94	0.73	0.51	0.94	0.52
MnBP					0.54	0.44	0.64	0.50	0.73	0.65	0.70
MCPP						0.24	0.54	0.43	0.51	0.56	0.48
MEP							0.36	0.28	0.38	0.36	0.33
MEHHP								0.78	0.54	0.98	0.55
MEHP									0.45	0.77	0.44
MiBP										0.55	0.60
MEOHP											0.56
MBzP											

*Correlations were statistically significant at $p < 0.0001$ level

Table S6. Overall Associations between Urinary Phthalate Metabolites and Metabolic Syndrome, NHANES (2005-2014).

Phthalate Metabolites	Multivariable[a] OR (95% CI)
**MCNP**	
**Q1**	1.00
**Q2**	1.09 (0.88, 1.36)
**Q3**	1.05 (0.82, 1.34)
**Q4**	0.97 (0.76, 1.23)
p for trend	0.67
**MCOP**	
**Q1**	1.00
**Q2**	1.18 (0.95, 1.47)
**Q3**	1.12 (0.89, 1.42)
**Q4**	1.25 (1.02, 1.54)
p for trend	0.06
**MECPP**	
**Q1**	1.00
**Q2**	1.01 (0.84, 1.23)
**Q3**	1.10 (0.90, 1.35)
**Q4**	1.26 (1.01, 1.59)
p for trend	0.03
**MnBP**	
**Q1**	1.00
**Q2**	1.10 (0.88, 1.40)
**Q3**	1.04 (0.83, 1.31)
**Q4**	0.93 (0.71, 1.21)

Phthalate Metabolites	Multivariable[a] OR (95% CI)
p for trend	0.50
**MCPP**	
**Q1**	1.00
**Q2**	0.89 (0.72, 1.11)
**Q3**	1.05 (0.85, 1.29)
**Q4**	1.14 (0.94, 1.40)
p for trend	0.09
**MEP**	
**Q1**	1.00
**Q2**	1.00 (0.82, 1.23)
**Q3**	0.96 (0.78, 1.18)
**Q4**	0.89 (0.70, 1.13)
p for trend	0.33
**MEHHP**	
**Q1**	1.00
**Q2**	1.04 (0.86, 1.26)
**Q3**	1.08 (0.88, 1.32)
**Q4**	1.15 (0.91, 1.45)
p for trend	0.23
**MEHP**	
**Q1**	1.00
**Q2**	0.84 (0.69, 1.02)
**Q3**	0.85 (0.67, 1.09)
**Q4**	0.71 (0.55, 0.90)
p for trend	0.02
**MiBP**	
**Q1**	1.00
**Q2**	1.04 (0.87, 1.25)
**Q3**	1.06 (0.85, 1.31)
**Q4**	1.17 (0.91, 1.50)
p for trend	0.25
**MEOHP**	
**Q1**	1.00
**Q2**	1.03 (0.85, 1.24)
**Q3**	1.05 (0.84, 1.30)
**Q4**	1.14 (0.89, 1.45)
p for trend	0.29
**MBzP**	
**Q1**	1.00
**Q2**	1.11 (0.90, 1.36)
**Q3**	1.08 (0.86, 1.36)
**Q4**	1.22 (0.96, 1.55)
p for trend	0.15

[a] Adjusted for age, urinary creatinine, sex, race, total caloric intake, education, smoking status, fasting status, and poverty level.

Quartile values (ng/ml): MCNP: Q1= 0.1- 1.2, Q2= 1.2- 2.4, Q3= 2.4- 4.7, Q4= 4.7- 730.3;

MCOP: Q1= 0.1- 3.7, Q2= 3.7- 8.4, Q3= 8.4- 23.2, Q4= 23.3- 3287.4; MECPP: Q1= 0.1- 9.6,

Q2= 9.7- 20.1, Q3= 20.2- 43.2, Q4= 43.4- 15828.0; MnBP: Q1= 0.2- 7.1, Q2= 7.1- 15.4,

Q3= 15.4- 31.4, Q4= 31.4- 25863.0; MCPP: Q1= 0.1- 1.1, Q2= 1.1- 2.4, Q3= 2.4- 4.9,

Q4= 4.9- 1588.7; MEP: Q1= 0.3- 26.9, Q2= 26.9- 74.6, Q3= 74.6- 240.7, Q4= 240.8- 31660.0;

MEHHP: Q1= 0.1- 5.8, Q2= 5.8- 13.1, Q3= 13.1- 29.3, Q4= 29.3- 9326.1; MEHP: Q1= 0.3- 0.6,

Q2= 0.6- 1.7, Q3= 1.7- 4.1, Q4= 4.1- 1966.1; MiBP: Q1= 0.1- 3.5, Q2= 3.5- 7.7, Q3= 7.7- 15.0,

Q4= 15.1- 14537.0; MEOHP: Q1= 0.1- 3.6, Q2= 3.6- 8.0, Q3= 8.0- 17.4, Q4= 17.4- 6079.9;

MBzP: Q1= 0.1- 2.5, Q2= 2.5- 6.0, Q3= 6.0- 13.9, Q4= 13.9- 16510.3

Table S7. Associations between Urinary Phthalate Metabolites and Metabolic Syndrome by Sex, NHANES (2005-2014).

Phthalate Metabolites	Men (n=5,060) Multivariable[a] OR (95% CI)	Women (n=4,957) Multivariable[a] OR (95% CI)
**MCNP**		
**Q1**	1.00	1.00
**Q2**	0.94 (0.67, 1.33)	1.19 (0.92, 1.54)
**Q3**	0.97 (0.67, 1.42)	1.05 (0.77, 1.43)
**Q4**	0.80 (0.57, 1.12)	1.12 (0.80, 1.56)
p for trend	0.24	0.70
**MCOP**		
**Q1**	1.00	1.00
**Q2**	1.19 (0.84, 1.69)	1.18 (0.87, 1.59)
**Q3**	0.98 (0.68, 1.43)	1.24 (0.85, 1.79)
**Q4**	1.04 (0.76, 1.41)	1.51 (1.17, 1.95)
p for trend	0.80	0.002
**MECPP**		
**Q1**	1.00	1.00
**Q2**	0.90 (0.68, 1.21)	1.10 (0.88, 1.39)
**Q3**	1.10 (0.82, 1.48)	1.04 (0.77, 1.40)
**Q4**	1.40 (0.99, 1.98)	1.07 (0.81, 1.42)
p for trend	0.02	0.76
**MnBP**		
**Q1**	1.00	1.00
**Q2**	1.24 (0.96, 1.60)	0.93 (0.70, 1.24)
**Q3**	1.00 (0.75, 1.34)	1.08 (0.79, 1.48)
**Q4**	1.03 (0.72, 1.48)	0.82 (0.56, 1.19)
p for trend	0.78	0.45
**MCPP**		
**Q1**	1.00	1.00
**Q2**	0.90 (0.67, 1.19)	0.88 (0.69, 1.13)
**Q3**	0.96 (0.72, 1.27)	1.14 (0.84, 1.55)
**Q4**	1.06 (0.76, 1.49)	1.25 (0.95, 1.64)
p for trend	0.57	0.06
**MEP**		
**Q1**	1.00	1.00
**Q2**	0.86 (0.63, 1.18)	1.18 (0.92, 1.50)
**Q3**	0.82 (0.62, 1.09)	1.13 (0.84, 1.51)
**Q4**	0.87 (0.64, 1.19)	0.91 (0.66, 1.26)
p for trend	0.38	0.60
**MEHHP**		
**Q1**	1.00	1.00
**Q2**	1.09 (0.80, 1.48)	0.98 (0.76, 1.26)
**Q3**	0.97 (0.72, 1.32)	1.17 (0.89, 1.53)
**Q4**	1.39 (1.04, 1.86)	0.89 (0.63, 1.26)
p for trend	0.04	0.73
**MEHP**		
**Q1**	1.00	1.00
**Q2**	0.83 (0.63, 1.09)	0.84 (0.63, 1.11)
**Q3**	0.92 (0.66, 1.29)	0.77 (0.57, 1.04)
**Q4**	0.88 (0.61, 1.26)	0.54 (0.38, 0.76)
p for trend	0.74	0.001
**MiBP**		
**Q1**	1.00	1.00
**Q2**	0.94 (0.70, 1.26)	1.12 (0.83, 1.52)
**Q3**	0.91 (0.67, 1.24)	1.17 (0.84, 1.62)
**Q4**	1.07 (0.73, 1.59)	1.24 (0.84, 1.83)
p for trend	0.79	0.26
**MEOHP**		
**Q1**	1.00	1.00

Phthalate Metabolites	Men (n=5,060) Multivariable[a] OR (95% CI)	Women (n=4,957) Multivariable[a] OR (95% CI)
**Q2**	1.20 (0.88, 1.63)	0.90 (0.72, 1.12)
**Q3**	1.04 (0.78, 1.39)	1.04 (0.78, 1.38)
**Q4**	1.48 (1.09, 2.01)	0.84 (0.60, 1.19)
p for trend	0.02	0.49
**MBzP**		
**Q1**	1.00	1.00
**Q2**	1.38 (1.08, 1.78)	0.87 (0.65, 1.17)
**Q3**	1.07 (0.81, 1.40)	1.11 (0.82, 1.51)
**Q4**	1.27 (0.96, 1.68)	1.19 (0.81, 1.76)
p for trend	0.42	0.23

[a]Adjusted for age, urinary creatinine, race, total caloric intake, education, smoking status, fasting status, and poverty level.

Quartile values (ng/ml): MCNP: Q1= 0.1- 1.2, Q2= 1.2- 2.4, Q3= 2.4- 4.7, Q4= 4.7- 730.3; MCOP: Q1= 0.1- 3.7, Q2= 3.7- 8.4, Q3= 8.4- 23.2, Q4= 23.3-3287.4;MECPP: Q1= 0.1- 9.6, Q2= 9.7- 20.1, Q3= 20.2- 43.2, Q4= 43.4- 15828.0; MnBP: Q1= 0.2- 7.1, Q2= 7.1- 15.4, Q3= 15.4- 31.4, Q4= 31.4- 25863.0; MCPP: Q1=0.1- 1.1, Q2= 1.1- 2.4, Q3= 2.4- 4.9, Q4= 4.9- 1588.7; MEP: Q1= 0.3- 26.9, Q2= 26.9- 74.6, Q3= 74.6- 240.7, Q4= 240.8- 31660.0; MEHHP: Q1= 0.1- 5.8, Q2= 5.8- 13.1, Q3= 13.1- 29.3, Q4= 29.3- 9326.1; MEHP: Q1= 0.3- 0.6, Q2= 0.6- 1.7, Q3= 1.7- 4.1, Q4= 4.1- 1966.1; MiBP: Q1= 0.1- 3.5, Q2= 3.5- 7.7, Q3= 7.7-15.0, Q4= 15.1- 14537.0; MEOHP: Q1= 0.1- 3.6, Q2= 3.6- 8.0, Q3= 8.0- 17.4, Q4= 17.4- 6079.9; MBzP: Q1= 0.1- 2.5, Q2= 2.5- 6.0, Q3= 6.0- 13.9, Q4= 13.9-16510.3

Table S8. Overall Associations between Urinary Phthalate Metabolite Summary Measures and Metabolic Syndrome, NHANES (2005-2014).

Phthalate Metabolites	Multivariable[a] OR (95% CI)
**∑ LMW**	
**Q1**	1.00
**Q2**	0.95 (0.78, 1.15)
**Q3**	0.94 (0.72, 1.21)
**Q4**	0.84 (0.67, 1.05)
p for trend	0.17
**∑ HMW**	
**Q1**	1.00
**Q2**	1.07 (0.90, 1.27)
**Q3**	1.26 (1.04, 1.53)
**Q4**	1.19 (0.97, 1.47)
p for trend	0.05
**∑ DEHP**	
**Q1**	1.00
**Q2**	1.11 (0.94, 1.33)
**Q3**	1.13 (0.92, 1.38)
**Q4**	1.24 (0.97, 1.58)
p for trend	0.10

[a]Adjusted for age, urinary creatinine, sex, race, total caloric intake, education, smoking status, fasting status, and poverty level.

∑ LMW: MEP, MnBP, and MiBP

∑ HMW: MCNP, MCOP, MCPP, MBzP, and DEHP (MEHP, MECPP, MEHHP, and MEOHP)

∑ DEHP: MEHP, MECPP, MEHHP, and MEOHP

Quartile values (ng/ml): ∑ LMW: Q1= 0.7- 48.3, Q2= 48.3- 116.8, Q3= 116.8- 298.5, Q4= 298.6- 31764.6; ∑ HMW: Q1= 1.6- 40.6, Q2= 40.6- 83.4, Q3= 83.4-168.8, Q4= 169.0- 32066.2; ∑ DEHP: Q1= 0.6- 20.6, Q2= 20.7- 44.3, Q3= 44.3- 94.6, Q4= 94.7- 32041.5

Table S9. Associations between Urinary Phthalate Metabolite Summary Measures and Metabolic Syndrome by Sex, NHANES (2005-2014).

Phthalate Metabolites	Men (n=5060) Multivariable[a] OR (95% CI)	Women (n=4957) Multivariable[a] OR (95% CI)
**∑ LMW**		
**Q1**	1.00	1.00
**Q2**	0.80 (0.58, 1.10)	1.13 (0.90, 1.43)
**Q3**	0.81 (0.57, 1.14)	1.05 (0.76, 1.46)
**Q4**	0.86 (0.61, 1.20)	0.82 (0.61, 1.10)
p for trend	0.43	0.23
**∑ HMW**		
**Q1**	1.00	1.00
**Q2**	0.87 (0.67, 1.14)	1.23 (0.97, 1.57)
**Q3**	1.09 (0.82, 1.46)	1.35 (1.02, 1.80)
**Q4**	1.17 (0.84, 1.64)	1.12 (0.86, 1.45)
p for trend	0.14	0.26
**∑ DEHP**		
**Q1**	1.00	1.00
**Q2**	1.24 (0.92, 1.66)	1.01 (0.81, 1.25)
**Q3**	1.13 (0.86, 1.48)	1.09 (0.81, 1.46)
**Q4**	1.57 (1.13, 2.17)	0.93 (0.67, 1.30)
p for trend	0.01	0.82

[a]Adjusted for age, urinary creatinine, race, total caloric intake, education, smoking status, fasting status, and poverty level.

∑ LMW: MEP, MnBP, and MiBP

∑ HMW: MCNP, MCOP, MCPP, MBzP, and DEHP (MEHP, MECPP, MEHHP, and MEOHP)

∑ DEHP: MEHP, MECPP, MEHHP, and MEOHP

Quartile values (ng/ml): ∑ LMW: Q1= 0.7- 48.3, Q2= 48.3- 116.8, Q3= 116.8- 298.5, Q4= 298.6- 31764.6; ∑ HMW: Q1= 1.6- 40.6, Q2= 40.6- 83.4, Q3= 83.4-168.8, Q4= 169.0- 32066.2; ∑ DEHP: Q1= 0.6- 20.6, Q2= 20.7- 44.3, Q3= 44.3- 94.6, Q4= 94.7- 32041.5
